# Analysis of the subsequent treatment of osteoporosis by transitioning from bisphosphonates to denosumab, using quantitative computed tomography: A prospective cohort study

**DOI:** 10.1016/j.bonr.2021.101090

**Published:** 2021-05-07

**Authors:** Koki Tsuchiya, Koji Ishikawa, Yoshifumi Kudo, Soji Tani, Takashi Nagai, Tomoaki Toyone, Katsunori Inagaki

**Affiliations:** a Department of Orthopaedic Surgery, Showa University School of Medicine, Tokyo, Japan; b Department of Orthopaedic Surgery, Yamanashi Red Cross Hospital, Yamanashi, Japan

**Keywords:** BMD, bone mineral density, BMI, body mass index, BTM, bone turnover marker, DXA, dual-energy X-ray absorptiometry scanning, eGFR, estimated glomerular filtration rate, RANKL, receptor activator of nuclear factor κB ligand, QCT, quantitative computed tomography, TRACP-5b, tartrate-resistant acid phosphatase 5b, total-P1NP, total N-terminal propeptide of type I procollagen, Osteoporosis, Denosumab, Bisphosphonates, Quantitative computed tomography, Subsequent treatment

## Abstract

**Purpose:**

Denosumab reduces bone resorption and improves bone mineral density (BMD). Studies have analyzed subsequent treatment transitioning from bisphosphonates to denosumab based on dual-energy X-ray absorptiometry scanning (DXA). Quantitative computed tomography (QCT) can help assess cortical and trabecular bones separately in three dimensions without the interference of the surrounding osteophytes. In the present study, we analyzed the subsequent treatment transition from bisphosphonates to denosumab using QCT.

**Methods:**

Thirty-two patients with postmenopausal osteoporosis to be treated with denosumab were recruited. The patients were divided into two groups (15 prior bisphosphonate and 17 naïve) based on their previous treatment. BMD of the lumbar spine and hip were evaluated by DXA and QCT at baseline and 12 months following denosumab treatment.

**Results:**

The percentage change in volumetric BMD assessed by QCT at 12 months significantly improved in the naïve group compared with that in the prior bisphosphonate group. The region-specific assessment of femur at 12 months revealed that denosumab treatment was effective in both cortical and trabecular bones except the trabecular region of the prior bisphosphonate group.

**Conclusion:**

Our study suggests that although denosumab treatment was useful in both treatment groups, BMD increase was significantly higher in the naïve group than in the prior-bisphosphonate group. Interestingly, in the prior-bisphosphonate group, denosumab treatment was more effective in the cortical region than the trabecular region. Our study offers insights into the subsequent treatment and permits greater confidence when switching to denosumab from bisphosphonates.

## Highlights

• We analyzed the treatment transition from bisphosphonates to denosumab using QCT.
• Percentage change in volumetric BMD at 12 months significantly improved in the naïve group.
• Denosumab treatment was more effective on the cortical region than the trabecular region.
• Our study offers insights into the subsequent treatment when switching to denosumab.

## 1 Introduction

Due to the rapidly aging population, the number of patients with osteoporosis has been increasing, and it is currently estimated to be 13 million in Japan (Yoshimura et al., 2009). Sustaining the benefits of a therapeutic agent for chronic conditions such as osteoporosis generally requires subsequent treatment. Bisphosphonates, the most commonly prescribed treatment for osteoporosis, have proven efficacy in preventing bone loss and fractures (Bone et al., 2004; Black et al., 2007; Black et al., 2000). Recent concerns about issues related to long-term bisphosphonate treatment have led to discussions on when to stop or switch the treatment (Shane et al., 2014). Thus, there is a clinical need to better understand the treatments following bisphosphonates and their safety profiles.

Denosumab blocks bone resorption leading to decreased bone turnover, increases BMD, and reduces fracture risk (Bone et al., 2017; Anastasilakis et al., 2017). A recent study, based on dual-energy X-ray absorptiometry scanning (DXA)-based 3D modeling, reported that denosumab was effective in both cortical and trabecular regions (Winzenrieth et al., 2018). Moreover, several studies have reported that switching oral bisphosphonates to denosumab improves lumbar spine BMD (LS-BMD) (Recknor et al., 2013; Roux et al., 2014; Suzuki et al., 2018; Nakamura et al., 2017; Ebina et al., 2018). However, these studies present limited information because DXA measurement provides only a two-dimensional areal BMD (2D-aBMD) that could be influenced by the occurrence of osteophyte formation (Diederichs et al., 2011). Quantitative computed tomography (QCT) allows separate measurement of cortical and trabecular bones as a three-dimensional volumetric BMD (3D-vBMD), without being influenced by degenerative change including osteophytes and calcification (Diederichs et al., 2011). As the cortical component has a prominent role in bone strength (Iolascon et al., 2013), a precise evaluation of region-specific BMD is relevant to understand the treatment effect.

The osteoblasts lose their bone-forming capacity in the presence of bisphosphonates, because bisphosphonates inhibit the differentiation and maturation of osteoblasts (Manzano-Moreno et al., 2015). Moreover, a previous study reported that the concentration of bisphosphonates is lower in the cortical bone than in the trabecular bone (Roelofs et al., 2012). Therefore, we hypothesized that the effects of denosumab may differ between the cortical and trabecular regions in patients with prior bisphosphonate treatment. The aim of this study was to assess the effects of denosumab on the cortical and trabecular bones in patients previously treated with bisphosphonates using QCT.

## 2 Materials and methods

### 2.1 Study design

The study was conducted with the approval of the ethics committee of the Yamanashi Red Cross Hospital and in accordance with the Declaration of Helsinki. Informed consent to participate in the study was obtained from each patient.

For this study, we prospectively enrolled patients with postmenopausal osteoporosis who were scheduled to start a single 60 mg subcutaneous dose of denosumab with the daily supplementation of vitamin D between November 2014 and November 2015 at our institution. Osteoporosis was diagnosed in accordance with the criteria established by the Japanese Society of Bone and Mineral Research (Soen et al., 2013). In our institution, we use eldecalcitol (activated vitamin D) 0.75 μg as a prophylactic drug for denosumab to avoid denosumab-induced hypocalcemia (Ishikawa et al., 2018a; Ishikawa et al., 2016). Patients were divided into the prior bisphosphonate group (prior-BP group) and naïve group based on whether they were previously treated with bisphosphonates. The sample size was calculated using Stat Flex version 6. According to previous reports (Kuroda et al., 2018; Ishikawa et al., 2018b), the effect size was set as 0.80, alpha error was set as 0.05, and beta error was set as 0.20. Power analysis indicated that 28 patients were needed for the study. Considering potential discontinuation, 32 patients were included in this study.

The present study comprised 15 patients with prior bisphosphonate treatment and 17 naïve patients. The inclusion criteria for the study were postmenopausal women older than 60 years with osteoporosis. The exclusion criteria were 1) secondary osteoporosis; 2) a history of diseases affecting the musculoskeletal system; 3) disorders such as uncontrollable thyroid disease and active malignant tumor; 4) a history of receiving medications that affect bone metabolism; or 5) a surgery in the past 6 months. The primary endpoint was to investigate the subsequent treatment response in cortical and trabecular regions using QCT. The secondary endpoint was to evaluate 2D-aBMD and bone turnover markers [BTMs: tartrate-resistant acid phosphatase 5b (TRACP-5b) and total N-terminal propeptide of type I procollagen (total P1NP)] in both treatment groups. All participants provided written informed consent, and the study was approved by the ethics committee of the Yamanashi Red Cross Hospital.

### 2.2 Data collection

We evaluated information from a baseline questionnaire, including age, weight, height, body mass index, previous fracture history, smoking history, alcohol consumption, and glucocorticoid use. We assessed laboratory data, DXA, and QCT measurements that may help evaluate the conditions of osteoporosis.

### 2.3 Biochemical measurements

The serum levels of albumin, calcium, and phosphorus, and estimated glomerular filtration rate (eGFR) were evaluated at baseline. The BTMs, TRACP-5b [estimated using the Osteolinks® TRACP-5b® Test Kit (DS Pharma Biomedical Co., Ltd., Osaka, Japan) according to the reference range in women (120–420 mU/dL)] and total P1NP [estimated using the total P1NP assay on Elecsys automated analyzer (Roche Diagnostics, Basel, Switzerland) according to the reference range in postmenopausal women (26.4–98.2 μg/L)] were assessed at baseline, and 1, 3, 6, 7, 9, and 12 months after treatment.

### 2.4 Bone mineral density assessed by dual-energy X-ray absorptiometry

2D-aBMD of the lumbar spine (LS-aBMD; L1–4), femoral neck (FN-aBMD), and total hip (TH-aBMD) were measured at baseline and 12 months following treatment using DXA (Hologic QDR series; Hologic, Waltham, MA, USA). All DXA measurements were analyzed by a radiologist at a central site.

### 2.5 Bone mineral density assessed by quantitative computed tomography

3D-vBMD of the lumbar spine (LS-vBMD), femoral neck (FN-vBMD), and total hip (TH-vBMD) were measured at baseline and 12 months following treatment. The details of the measurement of QCT have been described previously (Ishikawa et al., 2018b; Kuroda et al., 2018). CT data were acquired with a SOMATOM Definition AS+ multidetector-row CT scanner (Aquilion 16; Toshiba Medical Systems, Otawara, Japan) using predefined scanning conditions (x-ray energy, 120 kV; x-ray current, SD20; rotation speed, 0.5 s/rot; beam pitch, 0.95). A calibration phantom (Mindways, Austin, TX, USA) placed underneath the patients during each CT scan was used to convert the CT values to a BMD scale. The central 3D-BMD was analyzed using QCT-Pro software v4.1.3 with the QCT-Pro Bone Investigational Toolkit v2.0 (BIT; Mindways Software, Austin, TX, USA). Potential adverse effects of QCT exposure with the radiation dose approximately equal to abdomen and pelvic computed tomography were determined.

### 2.6 Statistical analysis

The χ^2^ test was used to compare categorical variables. Mann–Whitney *U* test was used to compare group means for non-normally distributed variables. ANCOVA (adjusted by baseline BMD) was performed to assess the percentage change in BMD from baseline between the groups. Wilcoxon signed-rank test was used to evaluate the longitudinal change in BMD and BTMs. The last observation carried forward method was used to analyze the data of one patient who did not appear for BTM evaluation at 9 months. The correlations between the percentage and absolute changes in BTMs and LS-aBMD were determined using Spearman's rank correlation coefficient. Statistical analyses were performed using Stat Flex version 6 (Artech, Tokyo, Japan) and EZR (Saitama Medical Center, Jichi Medical University, Saitama, Japan). All statistical tests were two tailed and results with *P*-values <0.05 were considered statistically significant.

## 3 Results

### 3.1 Study population

**Fig. 1:**
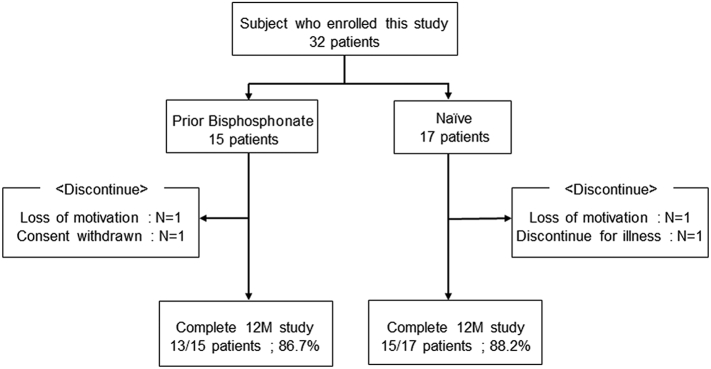
Study flow diagram.

Of the 32 patients enrolled in this study [15 prior bisphosphonate; 78.0 (69.8–82.0) years, 17 naïve; 74.0 (67.0–79.0) years], 13 patients (86.7%) in the prior-BP group and 15 patients (88.2%) in the naïve group completed the study (Fig. 1). There were no serious adverse events including fracture during the study period. The clinical characteristics of the patients are shown in Table 1. There were no significant differences in age, BMI, eGFR, calcium concentration, and BMD between the groups at baseline. The information of previous bisphosphonate treatment is summarized in Table 2. The mean duration of bisphosphonate usage was 18.2 months.

**Table 1:** Comparison of clinical parameters between the prior bisphosphonate and naïve groups.

Summary of the Parameters	Prior-BP (*n* = 15)	Naïve (*n* = 17)	P
Age	78.0 (69.8/82.0)	74.0 (70.0/79.0)	n.s
BMI	21.7 (20.7/23.5)	22.6 (20.5/25.5)	n.s
eGFR	68.0 (53.8/82.3)	67.0 (61.5/75.8)	n.s
Corrected Ca	9.1 (8.9/9.5)	9.1 (8.9/9.4)	n.s
History of previous fracture, n (%)	8 (53.3)	6 (35.3)	n.s
Current smoker, n (%)	2 (13.3)	1 (5.9)	n.s
Alcohol consumption, n (%)	1 (6.7)	0 (0)	n.s
Glucocorticoid use, n (%)	0 (0)	0 (0)	n.s

2D-Bone mineral density			
DXA: LS-aBMD (g/cm2)	0.74 (0.64/0.82)	0.71 (0.67/0.79)	n.s
DXA: FN-aBMD (g/cm2)	0.55 (0.49/0.56)	0.51 (0.49/0.58)	n.s
DXA: TH-aBMD (g/cm2)	0.65 (0.61/0.71)	0.69 (0.61/0.74)	n.s
DXA: LS-T score	−2.20 (−3.10/−1.55)	−2.50 (−2.83/−1.78)	n.s
DXA: FN-T score	−2.70 (−3.50/−2.48)	−3.10 (−3.33/−2.38)	n.s
DXA: TH-T score	−2.40 (−2.70/−1.70)	−2.80 (−2.95/−1.65)	n.s

3D-Bone mineral density			
QCT: LS-vBMD (g/cm3)	60.1 (46.5/83.0)	61.6 (49.8/74.2)	n.s
QCT: FN-vBMD (g/cm3)	236.3 (214.0/262.9)	240.1 (220.5/268.0)	n.s
QCT: TH-vBMD (g/cm3)	230.9 (216.6/262.8)	231.9 (200.7/264.0)	n.s

Bone turnover markers			
TRACP-5b (mU/dL)	308.0 (189.5/507.5)	534.0 (355.5/687.8)	0.021
Total-P1NP (μg/L)	29.6 (14.5/57.5)	53.6 (46.6/94.8)	0.012

Date shown as n or n (%) were analyzed by χ^2^ test.

Date presented as median IQR were analyzed by Mann-Whitney U test.

**Table 2:** Type and duration of previous bisphosphonate medication in the prior bisphosphonate groups.

Prior BP drug	N (%)	Duration (mean month)
Alendronate	11 (73.3)	17.2
Risedronate	2 (13.3)	7.5
Minodronate	1 (6.7)	12
Alendronate to Ibandronate	1 (6.7)	57 (54 to 3)

The dose of bisphosphonate:

Alendronate; 35 mg per week, Risedronate; 17.5 mg per week, Minodronate; 50 mg per month, Ibandronate; 100 mg per month.

### 3.2 Bone turnover markers

**Fig. 2:**
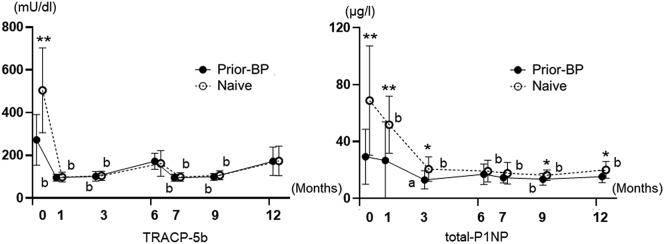
Changes in the serum TRACP-5b and total P1NP concentrations during the treatment period. Differences between the two groups were analyzed using Mann–Whitney *U* test. *P < 0.05, **P < 0.01. Longitudinal changes were analyzed using Wilcoxon signed-rank test. a: *P* < 0.05, b: *P* < 0.01.

BTMs (TPACP-5b, total P1NP) were significantly higher in the naïve group than in the prior-BP group at baseline (TRACP-5b: *P* < 0.05; total P1NP: *P* < 0.05) (Table 1). The changes in TRACP-5b and total-P1NP are shown in Fig. 2. The TRACP-5b and total-P1NP levels in both treatment groups significantly decreased from baseline, and they were suppressed until 12 months of treatment with denosumab.

**Fig. 3:**
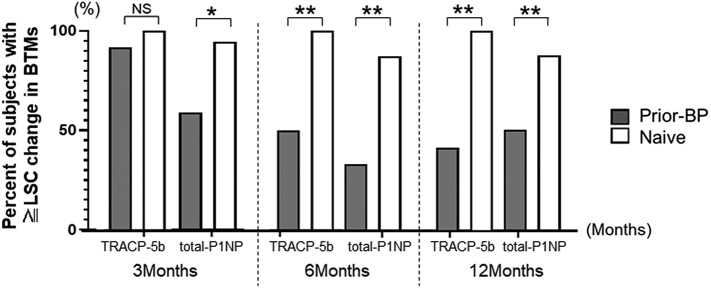
Percentage of subjects with ≥ least significant change (LSC) in BTMs at 3, 6, and 12 months. Differences between the two groups were analyzed using the χ^2^ test. *P < 0.05, **P < 0.01.

The longitudinal change in the TRACP-5b values significantly decreased in both treatment groups at 1, 3, 7, and 9 months. The longitudinal change in the total P1NP values significantly decreased in the naïve group at 1, 3, 6, 7, 9, and 12 months, and in the prior-BP group at 3 and 9 months (Fig. 2). Biochemical response to osteoporosis treatment can be assessed by observing the decrease in BTMs beyond the least significant change (LSC). According to previous reports, the calculated LSC in TRACP-5b was 34.3% and that in total P1NP was 38% (Nishizawa et al., 2019; Naylor et al., 2016). A significantly greater percentage of naïve subjects had decreased BTMs that were ≥ LSC during the treatment than prior bisphosphonate subjects [TRACP-5b (prior-BP group vs. naïve group) 6 months: 50% vs. 100%; *P* < 0.01, 12 months: 41.7% vs. 100%; *P* < 0.01; total P1NP (prior-BP group vs. naïve group) 3 months: 58.3% vs. 93.3%; *P* < 0.05, 6 months: 33.3% vs. 86.7%; *P* < 0.01, 12 months: 50% vs. 86.7%; *P* < 0.05]. This suggests that the chance of bone turnover was considerable in naïve subjects (Fig. 3).

### 3.3 Bone mineral density assessed by dual-energy X-ray absorptiometry

**Fig. 4:**
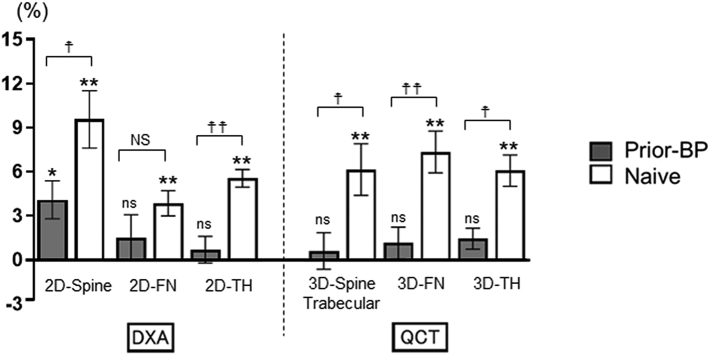
Percentage change in BMD from baseline to 12 months. Data are presented as LS mean and 95% CI. Longitudinal change was analyzed using Wilcoxon signed-rank test. *P < 0.05, **P < 0.01. Differences between the two groups were analyzed using the ANCOVA. ^☨^*P* < 0.05, ^☨☨^*P* < 0.01.

The longitudinal change from baseline to 12 months in 2D-aBMD measured by DXA was increased at each site in the naïve group but was increased only at the lumbar spine in the prior-BP group. The percentage change in LS-aBMD and TH-aBMD at 12 months was significantly higher in the naïve group than in the prior-BP group (LS-aBMD: *P* < 0.05; TH-aBMD: *P* < 0.01) (Fig. 4).

### 3.4 Bone mineral density assessed by quantitative computed tomography

The longitudinal change from baseline to 12 months in 3D-vBMD at each site measured by QCT was significantly higher in the naïve group than in the prior-BP group (LS-vBMD: *P* < 0.05; FN-vBMD: *P* < 0.01; TH-vBMD: *P* < 0.01) (Fig. 4).

The longitudinal change from baseline to 12 months in cortical TH-vBMD significantly increased in both treatment groups, but trabecular TH-vBMD did not increase significantly in the prior-BP group.

**Fig. 5:**
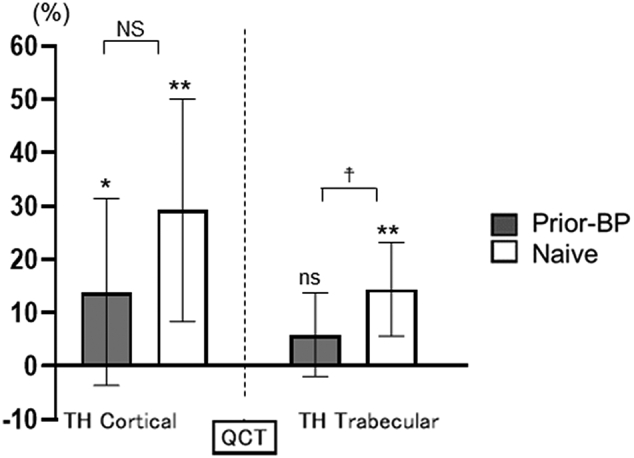
Percentage change in TH-BMD assessed by QCT from baseline to 12 months. Longitudinal change was analyzed using Wilcoxon signed-rank test. *P < 0.05, **P < 0.01. Differences between the two groups were analyzed using the ANCOVA with data presented as LS mean and 95% CI. ^☨^*P* < 0.05.

Compared with the percentage change in region-specific vBMD between the groups, trabecular TH-vBMD was significantly increased in the naïve group, whereas the changes were comparable in cortical TH-vBMD (Fig. 5). These results indicated that denosumab treatment was effective in the cortical and trabecular regions in the naïve group, but in prior-BP group, the treatment effect was inferior in the trabecular region than in the cortical region.

### 3.5 Correlations between the changes in bone turnover markers and bone mineral density

The response of BTMs following treatment can be useful to predict BMD (Zheng et al., 2015). We evaluated the correlation between BTMs and aBMD. Notably, in the naïve group, there were positive correlations between the absolute changes in BTMs at 1 month and 3 months and changes in LS-aBMD at 12 months. However, in the prior-BP group, there were no significant correlations between the changes in BTMs and LS-aBMD (Table 3).

**Table 3:** Relationships between the percentage and absolute changes in BTMs and LS-aBMD.

Percent change	Percent change in LS-aBMD				Absolute change	Absolute change in LS-aBMD			
	Prior-BP		Naïve			Prior-BP		Naïve	
	r	P	r	P		r	P	r	P
TRACP-5b(0-1 M)	−0.39	n.s.	−0.48	<0.05	TRACP-5b(0-1 M)	−0.22	n.s.	−0.55	<0.05
TRACP-5b(0-3 M)	−0.38	n.s.	−0.48	n.s.	TRACP-5b(0-3 M)	−0.24	n.s.	−0.55	<0.05
total-P1NP(0-1 M)	0.26	n.s.	−0.70	<0.01	total-P1NP(0-1 M)	0.041	n.s.	−0.71	<0.01
total-P1NP(0-3 M)	−0.08	n.s.	−0.48	n.s.	total-P1NP(0-3 M)	−0.16	n.s.	−0.72	<0.01

The correlations between the percentage and absolute changes in BTMs and LS-aBMD were determined using Spearman's rank correlation coefficient.

### 3.6 Safety profile according to hypocalcemia following denosumab treatment

**Fig. 6:**
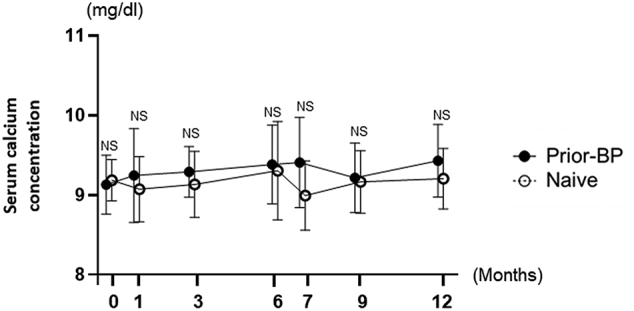
Changes in the serum calcium concentration during the treatment period. Data are expressed as mean ± SD; they were compared using Wilcoxon signed-rank test.

Fig. 6 shows the changes in albumin-adjusted serum calcium concentrations following treatment with denosumab over the treatment period. The serum calcium levels did not significantly change in either group. Hypocalcemia was defined as an adjusted serum calcium concentration, with 8.7 mg/dL as the lower limit of the normal range in our central laboratory. None of the patients were hypocalcemic at baseline, but 5 patients in the prior-BP group (33.3%) and 9 patients in the naïve group (52.9%) developed the Common Terminology Criteria for Adverse Events version 4.0. grade 1 asymptomatic hypocalcemia following treatment.

## 4 Discussion

In the present study, we investigated the effects of prior bisphosphonate treatment using QCT, a 3D technique that can be used to assess treatment effects on the cortical and trabecular bones separately. Our findings revealed that denosumab treatment was more effective for the cortical region than the trabecular region in patients who had received prior bisphosphonate treatment.

The results of our study showed that the BTM levels were higher in the naïve group than in the prior-BP group at baseline. However, although the baseline levels were different, BTMs reached similar levels of suppression at 12 months in both treatment groups. Therefore, denosumab has strong inhibitory effects on bone resorption, regardless of prior treatment with bisphosphonates, and this is consistent with the findings of a recent study (Kaneko et al., 2019).

BTMs can rapidly provide information on the early response to osteoporosis treatment. Moreover, the LSC is an important determinant in evaluating the changes in BTMs because it reflects the smallest change that, when equaled or exceeded, allows clinicians to conclude whether there has been a significant change in the measurement (Naylor et al., 2016; Roux et al., 2014). In the present study, the percentage of subjects with BTMs ≥ LSC was significantly higher in the naïve group than in the prior-BP group. Furthermore, there was a correlation between the early changes in BTMs and LS-aBMD in the naïve group. Thus, this result suggests that early changes in BTMs can predict treatment response in the naïve group. However, in the prior-BP group, the results of denosumab treatment could not be assessed by the lack of early BTM reduction.

In the present study, the serum calcium levels did not change significantly in either group, and this is consistent with the findings of previous studies (Nakamura et al., 2017; Kobayashi et al., 2020). However, we previously reported that a high bone turnover elevates the risk of denosumab-induced hypocalcemia (Ishikawa et al., 2018a; Ishikawa et al., 2016). Therefore, physicians should pay attention to hypocalcemia due to a strong suppression of BTMs in the naïve group.

In agreement with the findings of previous studies (Recknor et al., 2013; Roux et al., 2014), we found that the percentage change in LS-aBMD and TH-aBMD assessed by DXA at 12 months was significantly higher in the naïve group than in the prior-BP group. Moreover, the precise investigation using QCT in the present study confirmed that 3D-vBMD was significantly higher in the naïve group than in the prior-BP group. Notably, we demonstrated that TH-vBMD in the prior-BP group was significantly increased in the cortical bone but not in the trabecular bone.

These results may be explained by the effect of bisphosphonates on osteoblasts and the difference in the effects of the two agents on the cortical and trabecular bones. Bisphosphonates increase the degree of mineralization of the cortical and trabecular bones (Roschger et al., 2001), whereas the osteoblast loses its bone-forming capacity in the presence of bisphosphonates, because bisphosphonates inhibit the differentiation and maturation of osteoblasts (Manzano-Moreno et al., 2015). Moreover, bisphosphonate was detected in urine up to 19 months after the discontinuation of the drug (Peris et al., 2011). Collectively, bone formation may have decreased in the prior-BP group due to the accumulated bisphosphonate effect even after switching to denosumab.

In the trabecular bone, bisphosphonates and denosumab inhibit bone resorption similarly. Zebaze et al. showed that, in the cortical bone, denosumab circulates freely to bone surfaces and into remodeling compartments, where it inhibits osteoclastogenesis and thus inhibits remodeling more rapidly and considerably than bisphosphonates (Zebaze et al., 2014). Therefore, bisphosphonates are less likely to act on cortical bone structure than denosumab. In addition, a previous study showed that the concentrations of bisphosphonate are lower in the cortical bone than in the trabecular bone (Roelofs et al., 2012). Taken together, in the prior-BP group, denosumab treatment was effective in the cortical region of the total hip because the concentrations of bisphosphonates at which bone formation is reduces were lower in the cortical bone than in the trabecular bone. We recommend that clinicians should keep these results in mind in osteoporosis practice so that they can make more accurate decisions regarding subsequent treatment. Our study offers insights into the subsequent treatment and permit greater confidence when switching to denosumab from bisphosphonates.

The present study had some limitations. First, the number of patients was small, and the criterion for switching treatment and the indication of denosumab remained obscure. Second, given the fact that there was no significant difference in BMD at baseline between the groups despite a treatment history with bisphosphonate, the patients in the prior-BP group might have had severe osteoporosis. In addition, we did not assess the non-responders in the prior-BP group because most of the patients who switched from bisphosphonates lacked previous BMD data as they treated in general clinics. However, a previous study revealed that denosumab improves BMD and BTMs in bisphosphonate-unresponsive patients (Kamimura et al., 2017). Finally, we did not evaluate the biomechanical parameters. Therefore, our results might be insufficient for establishing the advantages of denosumab treatment.

## 5 Conclusions

In conclusion, we reported the effects of prior bisphosphonate treatment on the BMD using DXA and QCT in patients subsequently treated with denosumab. The present study demonstrated that although denosumab treatment was effective in both groups, BMD increase was greater in the naïve group than in the prior-BP group. Interestingly, in the prior-BP group, denosumab treatment was more effective on the cortical region than the trabecular region.

## CRediT authorship contribution statement

Research conception and design: Koji Ishikawa.

Data collection: Koki Tsuchiya, Koji Ishikawa, Soji Tani.

Interpretation of data: Koki Tsuchiya, Koji Ishikawa, Soji Tani.

Statistical analysis: Koki Tsuchiya, Koji Ishikawa.

Drafting the manuscript: Koki Tsuchiya, Koji Ishikawa.

Manuscript review: Koki Tsuchiya, Koji Ishikawa.

Study supervision: Yoshifumi Kudo, Takashi Nagai, Tomoaki Toyone, Katsunori Inagaki. Approval of the final manuscript: all the above-listed authors.

## Transparency document

Transparency document. Image 1

## Declaration of competing interest

None.
